# Indoor Radon Exposure Among Schoolchildren: A Systematic Review of Risk Factors

**DOI:** 10.3390/ijerph23060712

**Published:** 2026-05-27

**Authors:** Rasaq A. Yusuf, Thokozani P. Mbonane, Phoka C. Rathebe

**Affiliations:** Department of Environmental Health, Faculty of Health Sciences, University of Johannesburg, Doornfontein Campus, P.O. Box 524, Johannesburg 2006, South Africa; powerray2003@yahoo.com (R.A.Y.); tmbonane@uj.ac.za (T.P.M.)

**Keywords:** radon, indoor, schoolchildren, risk factors

## Abstract

**Highlights:**

**Public health relevance—How does this work relate to a public health issue?**

- As a Group 1 carcinogen and the leading cause of lung cancer among non-smokers, radon accumulation in schools poses a critical environmental health risk.
- Children spend a substantial amount of time in classrooms; exposure during key developmental stages can significantly increase their cumulative lifetime health risks.

**Public health significance—Why is this work of significance to public health?**

- This review synthesized evidence from various regions of the globe, thereby identifying geological factors, building structural defects, and ventilation-related factors that influence radon concentrations within educational buildings.
- While the average radon levels in this review frequently remain below 100 Bq/m^3^, there were some radon-prone regions on volcanic subsoil that exhibited extreme outliers surpassing 4000 Bq/m^3^, thereby necessitating mitigation measures.

**Public health implications—What are the key implications or messages for practitioners, policy makers and/or researchers in public health?**

- Public health agencies should establish regular radon testing and mitigation policies, with long-term monitoring and remediation in school buildings exceeding recommended reference levels.
- Policymakers should advance systematic radon risk management in schools by strengthening mitigation policies, including active soil depressurization, foundation sealing, improved ventilation, construction of radon-resistant school buildings, and mandatory radon testing.

**Abstract:**

Radon (222Rn) is a naturally occurring radioactive gas. It is colourless, odourless, and tasteless, produced through the spontaneous decay of uranium in soil and rocks. Among school-aged children, exposure to radon is a major public health concern because, during school hours, learners spend an average of 6–8 h daily inside school buildings, often on the ground floor or in basement classrooms, where radon levels tend to be highest. This study aims to contextualize radon exposure among children in educational settings, with a focus on the associated risk factors. A systematic review of the literature on radon exposure in classrooms among schoolchildren was conducted, analysing associated risk factors and methods of radon measurement. A literature search was performed across reputable databases to ensure compliance with systematic review standards. The quality of the evidence was appraised using the Grading of Recommendations Assessment, Development and Evaluation (GRADE) tool. A total of 32 studies met the inclusion criteria and were analyzed. Radon levels measured in classrooms exhibit variability based on geographic location. Certain classrooms in Continental Europe and North America exceed the WHO reference limit of 100 Bq/m^3^, as well as regional thresholds, including the European Union limit of 300 Bq/m^3^ and the United States Environmental Protection Agency (EPA) limit of 148 Bq/m^3^. Indoor radon exposure in classrooms is a worldwide concern because children are particularly vulnerable during their formative years. Those attending daycare centers and kindergartens are at greater risk due to their nascent respiratory systems.

## 1. Introduction

Radon (222Rn) is a naturally occurring radioactive gas that is colourless, odourless, and tasteless [1,2]. It is produced through the natural decay of uranium present in soil and rocks [3]. Radon exposure in schoolchildren is viewed as a major public health issue because of their still-developing respiratory systems [4,5,6]. Additionally, students usually spend an average of 6–8 h daily, and sometimes up to 10 h in certain settings, inside school buildings, often on the ground floor or in basement classrooms, where radon levels are typically higher [7,8,9].

Radon is identified as a Group 1 carcinogen by the World Health Organization (WHO) and the International Agency for Research on Cancer (IARC), backed by extensive scientific research [10,11,12]. It is the main cause of lung cancer in non-smokers and the second leading cause among smokers worldwide, with an estimated incidence rate of 3% to 14% [13,14]. In 2019, radon exposure was estimated to cause approximately 84,000 global lung cancer deaths, with the risk of developing the disease increasing by 16% for every 100 Bq/m^3^ increase in long-term exposure [11]. Although children are typically not classified as smokers and radon-associated health effects usually manifest after a considerable delay, children remain exposed to radon in environments such as in homes and educational institutions, thereby elevating the cumulative risk of developing lung cancer in later life.

This study focuses on radon exposure in schoolchildren due to a distinct challenge posed by their physiological and behavioral traits. For example, children’s respiratory rates, even when normalized for body weight, tend to be higher than adults’, leading to greater inhalation of radon [15,16]. Children’s lung epithelium shows increased radiosensitivity, with a longer latency before cancer develops [17]. Although evidence is still evolving, studies consistently associate childhood radon exposure to conditions such as childhood leukemia, exacerbation of asthma symptoms, and an increased dose–response relationship with inflammatory biomarkers [18,19,20,21,22].

An important perspective on pediatric radon exposure involves the significant amount of time children spend indoors at schools, especially in classrooms built on slab-on-grade foundations, in basements, or over crawl spaces, which tend to have higher radon levels [23,24,25]. Additionally, school ventilation and occupancy patterns—such as weekends when schools are closed and daily ventilation cycles—affect indoor radon levels in classrooms [26,27]. The risk increases further in resource-limited countries, where mechanical ventilation systems are seldom available. The geological context of the classrooms highlights an important issue: soils with high levels of uranium or radium often release significant radon amounts, frequently surpassing the thresholds set by the World Health Organization (100 Bq/m^3^) and the US EPA (148 Bq/m^3^) [28,29,30,31,32].

A thorough review of existing research was conducted to characterize indoor radon exposure among schoolchildren. The analysis examined radon concentrations in classrooms from different geographic areas and considered related risk factors. It included 32 studies, with evidence quality evaluated using the GRADE system. The review focused on answering the following key questions:

(a) What tools and methods are employed for radon measurement within classrooms?
(b) What are the measured indoor radon concentrations in schools across different regions?
(c) What risk factors contribute to increased radon exposure among schoolchildren?

## 2. Materials and Methods

### 2.1. Study Design

The study was conducted in accordance with the Population, Intervention, Comparison, and Outcome (PICO) framework. The framework facilitates comprehensive and systematic retrieval of relevant literature from multiple databases, improving sensitivity to various study types such as cross-sectional studies, national radon surveys, and systematic reviews [33,34,35]. As a result, it ensures consistent screening of publications and minimizes the chance of missing important studies.

This review employed a systematic scientific approach to examine radon exposure among schoolchildren, with a focus on the following:

Population (P): Children of school age, up to 18 years old.

Intervention (I): Exposure to indoor radon within classroom settings.

Comparison (C): Radon exposure levels among schoolchildren and the associated risks.

Outcome (O): Concentrations of indoor radon in classrooms.

### 2.2. Protocols and Registrations

This systematic review was conducted and reported in accordance with the Preferred Reporting Items for Systematic Reviews and Meta-Analyses (PRISMA) guidelines [33]. Ethical approval for the study was granted by the University of Johannesburg Faculty of Health Sciences Research Ethics Committee (REC; Clearance No. REC-2969-2024), and the review was registered with the International Prospective Register of Systematic Reviews (PROSPERO), under registration number CRD420261291315.

### 2.3. Information and Search Strategy

A systematic literature search was conducted across Scopus, Web of Science, PubMed/MEDLINE, Google Scholar, and the Cochrane Library to identify relevant peer-reviewed articles published up to January 2026. The search string utilized a combination of Medical Subject Headings (MeSH) terms and free-text keywords, focusing on quantitative studies.

The search strategy encompassed the following terms: (“Radon” OR “Radon gas” OR “222 Rn”) AND (“Classrooms”, “Schools” OR “Indoor” OR “Educational Facilities”) AND (“Schoolchildren” OR “Children”, “Pediatrics” OR “Radon in Children” OR “Children Exposure”) AND (“Risks” OR “Risk Factors”). The keywords were selected based on a preliminary scoping review and MeSH (Medical Subject Headings) terms to ensure maximum sensitivity. Subsequently, the reference lists of the retrieved articles were meticulously examined to identify relevant studies. The compilation of references for this study was uploaded to RAYYAN, a cloud-based platform specifically designed to assist researchers in conducting systematic reviews and meta-analyses [36].

### 2.4. Study Selection and Data Extraction Processes

#### 2.4.1. Study Selection and Data Extraction

The study’s Inclusion Criteria include the following: Population—school-age children less than 18 years old; selected studies—quantitative studies published in English language, including cross-sectional, cohort, systematic reviews, and panel studies; educational settings—daycare centers, kindergartens, primary and secondary schools; outcome—indoor (classroom) radon concentrations (Bq/m^3^ or pCi/L) with associated risk factor for radon exposure. Exclusion Criteria include the following: non-compliant environment—studies conducted exclusively within the workplace, or higher education centers (polytechnic, universities, etc.); adult population—studies targeting cohorts older than 18 years; unverified articles; editorial reviews; unpublished articles.

Data from the 32 identified studies were independently examined by three researchers using a standardized format to extract variables.

(a) Regional location of the study (Country);
(b) Study’s sample size;
(c) Duration of radon monitoring (short-term/long-term);
(d) Reported risk factors (soil geology, floor level, ventilation);
(e) Method of analysis engaged (Mean, Standard Deviation, Pearson’s correlation).

#### 2.4.2. Search Results

**Figure 1:**
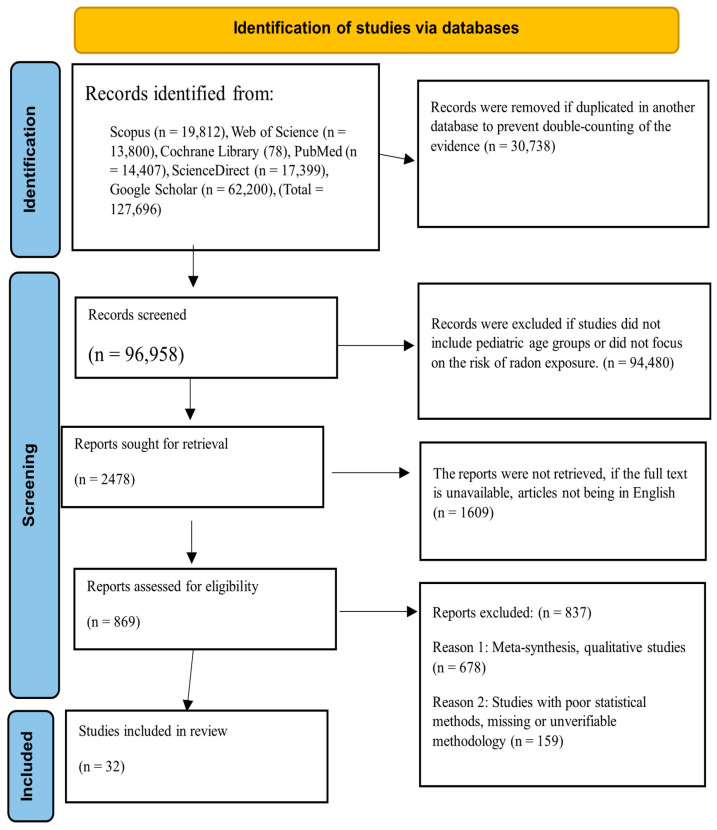
PRISMA Flow Chart on Literature Search.

A comprehensive search was conducted across multiple databases up until 28 January 2026, using the term “Radon,” which initially yielded 127,696 results. Duplicate records across databases were removed, reducing the number of articles to 96,958. To refine the search, we added specific keywords such as “Radon in Children,” narrowing the results to 63,301 articles. Further refinement involved adding “Risk Factors,” reducing the number to 2478. We then applied additional filters, selecting only published articles with full text and references, studies that included statistical analysis, peer-reviewed publications, and articles in English. By using the search terms “Radon,” “Exposure,” “Children,” and “Associate Risk factors,” connected by ‘OR,’ we retrieved a total of 869 articles. These were carefully reviewed, focusing on quantitative studies that examined radon exposure among school-age children under 18, related risks, methodological quality, and statistical analyses. Ultimately, 32 articles met our criteria and are detailed in Table S1 (Supplementary S1). Overall, this search process followed the PRISMA Flow Chart guidelines shown in Figure 1, ensuring a thorough and organized approach. We also included a complete PRISMA checklist as Table S2 (Supplementary S2).

#### 2.4.3. Rating the Quality of Evidence

This study evaluated the evidence regarding risk factors associated with radon exposure in schoolchildren through the GRADE framework. Although randomized controlled trials (RCTs) are generally regarded as the most rigorous form of research evidence, their application in Environmental Health is often limited by ethical concerns and the vulnerability of children, making observational and exposure-based studies necessary [37,38]. Consequently, this review primarily aggregated data from observational research. To uphold high-quality standards, a modified GRADE approach was employed, focusing on systematic reviews, cross-sectional, ecological, cohort, and panel study designs, which were rated according to the criteria in Table 1.

Due to the inherent limitations of environmental research, relying on observational study data is methodologically justified. When conducted with strict controls, cross-sectional, cohort, and case–control studies can serve as reliable alternatives to randomized controlled trials (RCTs), providing strong evidence for identifying correlations between exposures and associated risk factors [39,40]. To evaluate the quality of the 32 selected studies, the Newcastle–Ottawa Scale (NOS) was used. The assessment was based on each study’s specific design.

- Cohort and Case–control studies were evaluated using a scoring system that ranged from 0 to 9. Scores of 6 or higher indicated high quality, while scores from 4 to 5 were deemed moderate quality. Studies with scores below 4 were classified as low quality.
- Cross-Sectional Studies: Scores from 0 to 10 were assessed; scores of 7 or higher were deemed high quality, while scores between 5 and 6 indicated moderate quality. Studies scoring below 5 are classified as low quality. The evidence review quality of the 32 included studies is shown in Table 2.

#### 2.4.4. Risk of Bias Among Selected Studies

The methodological quality and potential bias of the 32 studies were assessed using the Newcastle–Ottawa Scale (NOS), with modifications for environmental cross-sectional studies and reviews. Overall quality was moderate to high (Table S1), but some domains had varying levels of risk.

Selection bias presented a concern in certain studies. For instance, research rated as “Moderate” on the NOS, such as [41]—a study conducted in Zone 1 areas of New York, and [42]—which utilized data from the Ural Mountainous region, employed convenience sampling from specific schools or regions identified as radon-prone areas, potentially leading to an overestimation of the average exposure for the entire national schoolchild population. Conversely, large-scale surveys, including those conducted by [43] and [44], exhibited minimal selection bias owing to their extensive, multi-regional sampling strategies.

Detection bias varies depending on the duration and timing of radon measurements. Short-term measurements (less than 3 months), such as those conducted by [45] and some of the data presented in [46], are particularly susceptible to temporal bias due to fluctuations in radon levels across seasons and during nighttime or weekend ventilation cycles. Furthermore, studies measuring radon during periods of non-occupancy, such as [5] (which involved continuous radon measurement), could exhibit some bias. Since radon tends to accumulate when buildings are sealed, failure to account for differences between classroom occupancy and unoccupied hours may result in an overestimation of health risks to schoolchildren.

Most studies concentrated on primary geological factors such as soil permeability and proximity to granite or volcanic deposits. However, some research highlighted a moderate confounding risk attributable to incomplete data concerning building characteristics. For instance, studies conducted by [47] and [48] identified ventilation as a risk factor but did not account for meteorological variables such as indoor humidity and temperature. Unlike in [49], these factors may influence radon levels.

#### 2.4.5. Dose Estimation Framework

Dose estimation in the reviewed studies was typically derived using the UNSCEAR or ICRP dose conversion framework:

E = C × F × O × DCF

where E = annual effective dose (mSv/year);

C = radon concentration (Bq/m^3^);

F = equilibrium factor between radon and progeny (commonly 0.4);

O = occupancy time (h/year);

DCF = dose conversion factor (approximately 9 nSv per Bq·h·m^−3^).

## 3. Results

### 3.1. Patterns of Indoor (Classroom) Radon Concentrations

Indoor radon measurement generally utilizes either passive or active techniques. Alpha Track Detectors (ATDs) are regarded as the benchmark for long-term exposure assessment owing to their cost-effectiveness and absence of power needs; however, they do not supply real-time data [5]. In contrast, Activated Charcoal (AC) devices enable swift and economical screening but are highly susceptible to humidity and only capture a final snapshot [41]. For analyzing temporal radon variations and assessing HVAC system performance, continuous radon monitors (CRMs) are suitable for acquiring high-resolution hourly data; however, CRMs require rigorous professional calibration to ensure optimal performance [41,46].

Records of elevated average concentrations were observed in Iberian schools, reaching 332 Bq/m^3^, with over 30% of classrooms exceeding the European Union reference level of 300 Bq/m^3^ [50]. A modeling study conducted across schools in the United Kingdom estimated radon concentrations exceeding 300 Bq/m^3^ [51]. Nonetheless, reported radon levels within European literature range from 6 to 1478 Bq/m^3^ [43]. Moderate levels, nearing the guidelines established by the World Health Organization (100 Bq/m^3^), were documented in the following countries: China (84.3 Bq/m^3^)—[52]; Finland (86 Bq/m^3^)—[53]; Ireland (93 Bq/m^3^)—[54]; and Italy (77 Bq/m^3^)—[55].

Lower mean radon concentrations (<50 Bq/m^3^) were documented in Iran, Thailand, Kuwait, and parts of the United States [9,48,56,57], although localized high readings were still detected in specific buildings.

Slovenian surveys reported ranges of 70–770 Bq/m^3^ in schools and 145–794 Bq/m^3^ in kindergartens [58]. Finnish measurements reached maxima of 4205 Bq/m^3^ [53], Russian surveys recorded peaks at 620 Bq/m^3^ [42], and radon-prone Belgian schools frequently exceeded 400 Bq/m^3^ [59].

Among the studies meeting the inclusion criteria, reported annual effective doses were identified, ranging from 0.07 to 1.18 mSv/year in Spanish schools [60]; 0.3 mSv/year in Ireland [54]; and 0.4–0.48 mSv/year in Kuwaiti schools [48].

The reported geometric mean of radon concentration in Kuwait ranged from approximately 16 to 19 Bq/m^3^ [48], whereas in Israel, levels exceeded 10,000 Bq/m^3^ prior to mitigation measures [25].

### 3.2. Observed Determinants of Radon Levels

The majority of educational institutions situated on soils that are rich in uranium and radium, including areas with granitic bedrock, volcanic subsoils, permeable geological formations, tectonic fracture zones, and regions exhibiting high soil-gas permeability, have demonstrated elevated radon levels [42,55,58,60,61]. Moreover, these facilities located within radon-prone areas have shown both increased mean radon concentrations and significant outliers, with some measurements exceeding 4000 Bq/m^3^ [53], and levels surpassing 10,000 Bq/m^3^ prior to the implementation of mitigation strategies [25].

Building design and structural integrity influence radon ingress. Consistent reports across thirty-two studies indicated that ground-floor classrooms, slab-on-grade construction, basement spaces, structural cracks, floor–wall joints, utility penetrations, deteriorating infrastructure, stone foundations, and buildings constructed prior to radon-resistant standards were repeatedly associated with elevated concentrations [25,45,50,62]. Rooms in contact with the ground were documented to have higher radon concentrations compared to upper floors [51,52]. Inactive or inadequately maintained ventilation systems, the absence of mechanical ventilation, reduced air exchange during nights, weekends, and holidays, as well as insufficient classroom air exchange rates contributed to the accumulation of radon [5,46,49,55,56,58].

The indoor environmental conditions were correlated with the accumulation dynamics of radon. Physical parameters, including ambient temperature, relative humidity, and barometric pressure differentials, influenced both the rate of radon ingress and its atmospheric persistence [63,64]. Thermal gradients between the building envelope and the sub-slab environment frequently induced a convective ‘stack effect,’ particularly during the heating season, thereby facilitating pressure-driven radon entry. Moreover, ventilation cycles, characterized by school closures and occupancy-dependent HVAC adjustments, exacerbated temporal variability. Specifically, reduced air exchange rates during non-occupancy periods resulted in a ‘rebound’ effect in radon concentrations [5,55,59].

Furthermore, methodological challenges, such as short-term measurement volatility and spatial heterogeneity, complicate the accurate characterization of long-term chronic exposure [46,51,63]. These technical hurdles were compounded by institutional and policy-level deficiencies. The absence of comprehensive mandatory testing frameworks in certain educational settings, along with fragmented surveillance and a lack of standardized enforcement protocols, impeded systematic risk mitigation and public health management [44,65,66].

### 3.3. Radon Measurement Metrics

Passive radon detectors, including alpha track detectors (ATDs) such as CR-39 solid-state nuclear track detectors and electret ion chambers, were installed within educational institutions to measure radon concentrations [5,45,53,54,58,61,67]. These instruments were operational for durations ranging from two to twelve months. Furthermore, short-term radon measurement devices were employed for rapid screening, methodological validation, or preliminary assessment; these included activated charcoal canisters, continuous radon monitors (CRMs), and electronic radon detectors [41,44,46,56].

Some of the environmental surveys incorporated seasonal or multi-phase sampling designs to capture temporal variability. For example, ref. [63] examined seasonal variability, while [5] measured radon levels continuously during occupied and non-occupied periods. Spatial sampling strategies were also employed to characterize intra-building variability. Detectors were deployed across multiple rooms and floors to compare ground-floor versus upper-floor concentrations, assess basement classrooms, and implement multi-room sampling grids [51,52,64]. Statistical modeling approaches were utilized to characterize radon distributions and enhance exposure estimation. Techniques included modeling of log-normal distributions, estimation of radon distribution parameters, and predictive exposure modeling [51].

Large-scale national surveys employed standardized protocols for radon testing and reporting. For example, ref. [44] conducted protocol-based national assessments, while ref. [65] analyzed testing practices and prevalence. Systematic reviews compiled radon measurements and exposure factors across multiple studies [43,49]. These studies, employing meta-analyses, determined radon concentration ranges, exposure estimates, and risk factors from the existing literature.

Radon exposure within educational settings was assessed using a combination of methodologies, including passive dosimetry, active monitoring devices, short-term screening, statistical modeling, and survey evaluations, as delineated in Table 3.

## 4. Discussion

### 4.1. Indoor Radon Concentrations in Educational Settings

An analysis of 32 studies indicates that indoor radon concentrations within schools and other educational institutions exhibit considerable variation based on geographical location, temporal factors, and regional characteristics. These fluctuations are influenced by natural radon sources, architectural features, and ventilation systems. Although the mean radon levels typically remain beneath the World Health Organization’s threshold of 100 Bq/m^3^, the distribution of data is frequently right-skewed, with certain areas surpassing safety limits and presenting significant outliers. This underscores the importance of not relying exclusively on average values, as they may fail to identify localized environments with elevated risk. The geological context largely predicts indoor radon levels in schools. Studies show that schools on uranium-rich, granitic, or volcanic soils often have high radon. Branco et al. [50] noted 32.7% of Iberian schools exceeded 300 Bq/m^3^, linked to granitic soil. Similarly, ref. [53] reported peaks of 4205 Bq/m^3^ in Finland due to porous soil.

European studies have documented some of the highest variability and exceedance frequencies, particularly in regions characterized by granitic or volcanic geology, which are known to enhance radon emission and soil gas migration [43,50]. Conversely, investigations conducted in certain parts of Asia and the Middle East generally demonstrated lower average levels, attributable to differences in soil composition and building construction; nevertheless, localized elevated readings in ground-contact classrooms emphasize that regional averages do not negate exposure risks [9,48]. Studies in North America observed moderate levels but also exhibited substantial variation in testing coverage and mitigation strategies, implying that exposure may be underestimated in areas lacking testing [44,65]. Data from sub-Saharan Africa are scarce, limiting regional inferences; however, existing data indicate relatively low average levels alongside significant monitoring gaps [45].

Variability within buildings is consistently observed. Elevated radon concentrations in ground-floor and basement classrooms indicate proximity to soil gas entry points and pressure-driven infiltration pathways [52,62]. This spatial pattern is consistent with the physics of radon transport, wherein pressure differences between indoor air and soil gas facilitate radon ingress through foundation interfaces. Furthermore, the log-normal distribution of indoor radon levels suggests that only a few rooms may pose significant exposure risks, emphasizing the importance of room-specific measurements rather than reliance on building-wide averages [51].

Temporal variability complicates the assessment of radon exposures. Elevated radon levels observed at night, during weekends, and amidst school closures are associated with diminished ventilation and reduced air exchange rates, thereby facilitating the accumulation of radon [5,55]. Seasonal fluctuations, especially in winter, are attributable to increased building sealing and an enhanced stack effect, which amplify soil-gas infiltration [63]. These observations indicate that radon exposure varies over time rather than remaining static, highlighting the importance of conducting long-term evaluations to precisely characterize exposure profiles.

Although students spend less time in school than in residential settings, their cumulative exposure over multiple years remains considerable. Children may accumulate more than 10,000 h in classrooms throughout their compulsory education, and repeated exposure during critical developmental stages could heighten the risk of long-term lung cancer [8,68]. In this review, most effective annual dose estimates complied with international safety standards; however, some approaches or recommended thresholds in high-radon environments indicated that educational settings can significantly contribute to overall lifetime radon exposure.

Children in daycare and kindergartens are especially vulnerable to radon due to their developing lungs and higher breathing rates. These facilities, often on ground floors or basements, are in contact with soil-gas entry points. Evidence from the reviewed studies shows ground-level and basement classrooms have higher radon levels. For example, surveys in Slovenia and Finland reported radon levels up to 794 Bq/m^3^ and 2426 Bq/m^3^ [53,58]. In Israel, basement classrooms had up to 10,000 Bq/m^3^ [25], mainly due to uranium-rich soil and structural cracks. These findings highlight the need for mandatory testing and mitigation to protect children.

### 4.2. Mechanisms of Radon Ingress and Exposure Risk

The evidence indicates that radon exposure in educational settings arises from a complex, multilevel risk framework involving geological, structural, environmental, operational, and institutional factors. Comprehending these interconnected influences is vital for implementing targeted mitigation strategies and reducing associated risks.

In this review, geogenic factors serve as the primary upstream determinants of radon potential. Soils containing uranium and radium, along with granitic bedrock, volcanic formations, and tectonic fault systems, facilitate radon production and migration [58,60,61]. Furthermore, elevated soil permeability enhances radon transport by permitting soil gas to infiltrate building foundations. Educational institutions located in radon-prone regions consistently exhibit the highest concentrations and anomalies, thereby underscoring the pivotal influence of geological factors.

Building design and structural integrity are pivotal factors influencing radon ingress. Surfaces in contact with the ground, slab-on-grade constructions, basements, and foundation cracks function as pathways for soil gas infiltration [25,44]. Older edifices are particularly vulnerable due to deterioration and the absence of radon-resistant construction techniques [45,47]. Moreover, stone and granite materials may emit radon, thereby elevating indoor concentrations [67]. These structural deficiencies elucidate why heightened radon levels frequently manifest in specific rooms rather than throughout the entire structure. This aligned with the findings of [52], with exceedances mainly in ground-floor classrooms. Structural issues also matter as was pointed out by [61] who reported 4% of Brazilian classrooms exceeded 300 Bq/m^3^ due to cracks and openings. Older buildings are at higher risk as alluded by [55] with the observation of higher concentrations in older Italian schools. Conversely, ref. [45] suggested the low (4%) exceedance in South Africa might be due to concrete slab foundations acting as barriers.

Ventilation performance is a significant modifiable factor influencing indoor radon levels. Poorly maintained HVAC systems, lack of mechanical ventilation, and reduced air exchange during nights and closures have been consistently associated with higher radon concentrations [46,56]. The inverse relationship between the air exchange rate and radon levels demonstrates dilution effects: enhanced ventilation diminishes radon accumulation by replacing indoor air with outdoor air. Elevated radon levels observed during COVID-19 closures served as a natural experiment confirming the role of ventilation in reducing exposure [55].

Environmental factors additionally influence the ingress of radon. Variations in temperature, humidity, and seasonal fluctuations in atmospheric pressure impact the depressurization of buildings and the flow of soil gases [69]. During winter, the use of heating intensifies indoor–outdoor pressure differentials, thereby augmenting radon infiltration via the stack effect [63].

Operational factors, including patterns of room utilization and occupancy cycles, influence fluctuations in exposure over time. During periods when classrooms are closed and unoccupied (e.g., overnight or on weekends), in the absence of ventilation, soil gas tends to accumulate; however, the health risk remains negligible because no individuals are present [5,46]. Radon concentrations may increase, particularly in areas with insufficient ventilation; nonetheless, the actual exposure dose depends on both concentration and duration. Consistent airflow, such as through open windows or cross-ventilation, is highly effective in diluting radon to approximately 10–15 Bq/m^3^, aligning with near-ambient outdoor levels, thereby substantially mitigating health risks [58]. Moreover, assigning younger children to ground-floor classrooms may inadvertently elevate their exposure risk due to the proximity of these rooms to radon entry points. Kitto [41] showed that radon levels in New York schools were often lower than in nearby residences due to active HVAC during school hours. In areas with less mechanical ventilation, natural ventilation plays a key role; [47] found radon levels in Jordan were influenced by building age and ventilation habits, like opening windows. Also, ref. [57] noted that generally low radon levels in Thailand are due to effective cross-ventilation in school designs.

At the systemic level, policy and institutional determinants substantially affect the ability to detect and mitigate issues. The absence of obligatory testing protocols, fragmented monitoring efforts, and insufficient technical training all play a role in the under-detection of elevated radon levels [65,66]. These governance deficiencies are particularly consequential in low-resource settings where surveillance infrastructure is limited. Hence, effective radon prevention in schools requires mandatory testing and systematic risk management. Key measures include soil depressurization, improved ventilation, and radon-resistant building standards. Public health agencies should implement regular testing and long-term monitoring, especially for buildings above reference levels. Policymakers need to provide training and enforcement to ensure remediation in high-risk schools.

**Figure 2:**
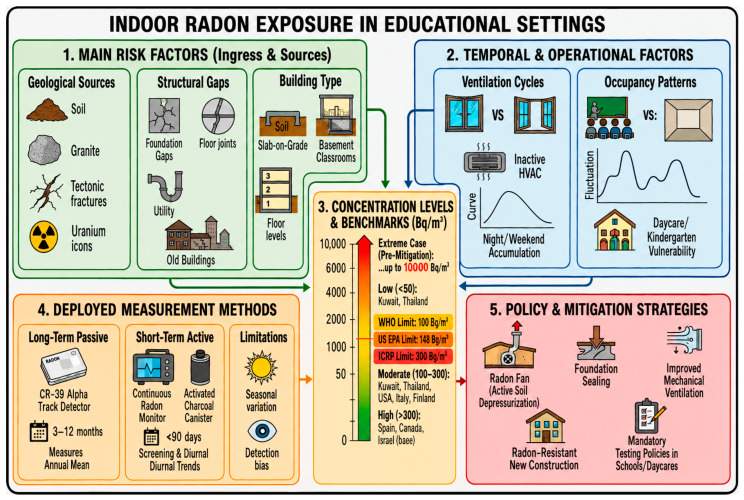
Schematic summarizing Indoor Radon Exposure in Educational Settings.

Collectively, the evidence supports a hierarchical risk model whereby geological potential establishes the baseline risk; structural features facilitate radon entry; ventilation regulates its accumulation; environmental factors affect transport; and policy measures determine the effectiveness of detection and mitigation as shown below in Figure 2.

### 4.3. Deployed Methods of Radon Measurement Within Classrooms

The studies examined employed various measurement methods, indicative of differing study objectives, technical expertise, and regulatory contexts. Nevertheless, a discernible pattern in methodologies is observed, with long-term passive monitoring emerging as the most dependable approach for the assessment of exposure.

Passive long-term detectors such as alpha track detectors, CR-39 detectors, and electret ion chambers are among the most commonly utilized instruments [5,45,67]. These devices assess radon exposure over extended periods (typically 3–12 months), capturing seasonal variations and generating averages that represent annual exposure levels. Their cost-effectiveness, dependability, and independence from electrical power sources render them ideal for large-scale monitoring and epidemiological investigations. The detection methodology, which involves the analysis of alpha-particle tracks resulting from radon progeny decay, offers a cumulative record of exposure, thereby enhancing measurement stability. Studies using these methods, such as [54] and [50], are classified as High-Yield due to their long-term data collection, which meets the gold standard for evaluating environmental health risks in vulnerable groups, such as schoolchildren.

Short-term measurement methods, such as activated charcoal canisters and electronic radon detectors, were employed for rapid screening and initial assessments [46,56]. These methods yield immediate results and are helpful for diagnostic purposes; however, their sensitivity to short-term variations restricts their effectiveness in estimating annual exposure. Studies that use short-term “snapshots” (less than 3 months), like [42], tend to be more affected by temporal bias. Factors such as mechanical ventilation, occupancy patterns, and weather conditions can significantly affect short-term readings, potentially leading to misclassification of exposure risk. The variability across studies highlights the need for long-term monitoring to assess chronic exposure. High-yield studies like [54] used standardized protocols and long-term passive dosimetry for reliable national exposure estimates. These differ from short-term “snapshot” screenings, which, though quick, may produce inconsistent results because of seasonal and daily changes.

Continuous radon monitors yielded high-resolution temporal data, facilitating the evaluation of diurnal variations and fluctuations associated with ventilation [46,55]. These devices exhibited significant increases in concentration during intervals of diminished ventilation, underscoring the critical role of air exchange in managing exposure. Continuous monitoring proves particularly valuable for pinpointing peak exposure periods and assessing the efficacy of mitigation measures.

Seasonal and multi-phase sampling methodologies improved exposure assessment by considering temporal variations such as stack effect associated with weather conditions and building utilization [63]. Hence, refs. [63] and [64] postulated that seasonal thermal gradients can induce a convective stack effect during the heating season, thereby facilitating pressure-driven radon entry into buildings. Sampling across various rooms and floors revealed significant within-building variability and demonstrated that classrooms in contact with the ground typically exhibit higher concentrations [52,62]. These findings underscore the insufficiency of singular measurements and support the implementation of room-level monitoring.

Statistical modeling techniques have further advanced exposure assessment by demonstrating that indoor radon concentrations adhere to a log-normal distribution [51]. Methods that incorporate distribution parameters and geometric means provide more accurate exposure estimates than basic averages, particularly within heterogeneous indoor environments.

National surveys and protocol-based assessments offered valuable insights into testing coverage, compliance, and exposure patterns, rather than functioning as primary measurement data [44,65]. Case studies that integrate geological and structural assessments provided essential insights into extreme exposure scenarios and the efficacy of mitigation strategies [25,58].

Overall, the diversity of methodologies observed across studies reflects varying surveillance objectives; however, the evidence robustly indicates that long-term passive monitoring constitutes the most dependable approach for assessing chronic exposure. Continuous monitoring, spatial sampling, and statistical modeling each provide significant insights into how exposure fluctuates over time and varies across different locations. The use of standardized measurement protocols and extended monitoring durations of 3 months or more are the essentials for accurately characterizing exposure levels and effectively managing radon-related risks in educational environments.

### 4.4. Strengths and Limitations of the Study

#### 4.4.1. Strengths

A significant strength of this systematic review lies in its global scope and comprehensive synthesis, incorporating 32 studies from diverse continents and a range of geological, climatic, and infrastructural contexts. This extensive scope enhances the external validity of the findings and facilitates the identification of consistent exposure patterns and risk factors transcending regional variations.

The review encompasses studies characterized by moderate to high methodological quality, including national surveys, environmental monitoring initiatives, and long-term dosimetry investigations [34,53,67]. The extensive adoption of standardized radon measurement protocols, guided by the WHO, EPA, and ICRP, enhances the comparability and dependability of the exposure data [21,25].

Another significant strength is the adoption of a multi-faceted analytical approach in synthesizing evidence. Rather than limiting the review solely to concentration levels, the study incorporates:

- Radon exposure distributions;
- Annual effective dose estimation;
- Structural and environmental determinants;
- Methodological approaches to measurement;
- Policy and institutional frameworks.

This systems-level synthesis offers a comprehensive understanding of radon exposure patterns within educational environments and supports the formulation of evidence-based strategies for risk mitigation [9,53,58].

The review further emphasizes intra-building variability and microenvironmental exposure differences, an aspect of radon research that is frequently neglected. By concentrating on spatial gradients (e.g., ground-floor versus upper-floor classrooms) and temporal variations in ventilation and occupancy patterns, the study advances understanding of exposure dynamics beyond mere average concentration reporting [9,52,62].

The review emphasizes child-specific exposure concerns, such as cumulative exposure during critical developmental periods and heightened physiological susceptibility to ionizing radiation [5,54]. This focus underscores the public health importance of the findings and aligns with risk-based environmental health frameworks.

Finally, the synthesis identifies modifiable factors, particularly ventilation performance and the integrity of the building–ground interface, providing practical insights for policymakers, school administrators, and environmental health professionals [46,64,66].

#### 4.4.2. Limitations

Despite this study’s strengths, certain limitations should be considered when interpreting the findings.

The review is constrained by notable disparities among the selected studies, such as differences in measurement duration, types of radon detectors, sampling methodologies, seasonal timing, and reporting metrics [41,46,61]. Furthermore, it is acknowledged that the studies were not conducted within a singular educational setting but across various environments, including daycares, kindergartens, schools, and mixed settings [9,13,58].

The distribution of geographic data is uneven, characterized by an overrepresentation in Europe and North America, whereas data from Africa, Latin America, and certain regions of Asia are limited [42,43,45,50,55]. This imbalance restricts the global applicability of the findings and underscores the need to improve radon monitoring initiatives in regions that are underrepresented.

Variability in measurement methods can lead to exposure misclassification. Short-term measurements [41,44], employed in certain studies, may not accurately represent annual exposure owing to fluctuations caused by ventilation, occupancy, and weather conditions. Variations in detector placement, seasonal sampling, and room selection further augment measurement uncertainty.

## 5. Conclusions

This review evaluates indoor radon exposure within educational institutions and its associated risk determinants. Radon concentrations demonstrate significant variability based on geographic location, building classification, and operational factors [41,45,49,54,61]. Although the majority of measured radon levels remain below the EPA, ICRP, and WHO thresholds [45,48,63,64,70], instances of exceedance are frequently observed in high-risk regions and vulnerable structures [43,50,59]. Concentrations surpassing 300 Bq/m^3^, and occasionally reaching into the thousands [42,53], underscore high-risk zones that necessitate targeted monitoring and intervention.

Radon exposure in educational institutions is influenced by various determinants. The geological substrate establishes the baseline potential, whereas building design, construction, and ground contact facilitate radon ingress [53,56,58,69]. Ventilation effectiveness is essential; inadequate air exchange results in accumulation. Environmental variables, seasonal fluctuations, occupancy patterns, and classroom locations further influence exposure levels. Policy deficiencies, such as the absence of mandatory testing and fragmented monitoring systems [44,70], impede prompt detection and effective mitigation.

Children experience repeated radon exposures during critical developmental periods; even though they spend fewer hours in school than at home, elevated concentration levels in certain educational buildings contribute significantly to their total exposure doses. The cumulative radon exposure over the years has substantial epidemiological significance, as it is associated with an increased risk of lung cancer in later life [21,68]. Furthermore, emerging research has tentatively linked exposure to other pediatric health issues, such as childhood leukemia [20,22]. Although estimates of effective dose within educational settings generally remain within public safety limits [48,55], they can approach or exceed thresholds in high-radon environments, underscoring the importance of monitoring and possible mitigation interventions.

## Figures and Tables

**Table 1:** Factors Affecting Evidence Review Quality [22].

	High-Yield Qualities	Low-Yield Qualities
Study Sample Size	Sample size of at least 300	Sample size less than 300
Findings	Consistent findings	Inconsistent findings
Confounders	Confounders controlled within the study	Confounders not controlled
Study Type	Systematic review, cohort, case–control, and panel studies	Cross-sectional studies, case studies
Duration of Classroom Radon Testing	Long-duration-radon measurement ≥90 days	Short-duration radon measurement ˂90 days
Study Location Selection	Randomized selection of study sites	Convenience sampling, e.g., radon-prone sites, radon zone 1
Peer-reviewed Publications	Peer-reviewed articles	Not peer-reviewed
Method of Radon Sampling	Alpha track detectors (ATDs) or electret ion chambers (EICs) for radon measurement >90 days	Electronic radon detectors, continuous radon monitors (CRMs), real-time electronic sensors—short-duration radon measurement, usually <90 days

**Table 2:** The evidence review quality of the 32 included studies.

Grouping	Description	Included Studies	Justification
Consistent Findings (High-Yield)	Results align with universal principles of radon ingress, including geological drivers and ground-floor risks.	[41,42,43,44,45,46,47,48,49,50,51,52,53].	Large-scale or long-term (>90 days) studies with >300 participants confirm soil-gas permeability and ground contact as key exposure factors.
Inconsistent Findings (Low-Yield)	Results show significant temporal variability or deviations from patterns due to study design.	[5,9,25,54,55,56,57,58,59,60,61,62,63,64].	Using short-term snapshot monitoring, mixed-method or convenience sampling can overestimate long-term exposure or overlook seasonal “rebound effects.”
Reviews/Meta-Data	Studies focusing on secondary data analysis or qualitative synthesis.	[65,66,67,68,69,70].	Peer-reviewed, synthesized from existing data, and/or offer conceptual consistency without new primary measurements.

**Table 3:** Radon Exposure in Educational Settings: Evaluation Methods.

Radon Assessment	Tools and Methods Deployed for Radon Measurement	Deployment Duration	Key Research Applications & Citations
Long-Term Passive Measurements	Alpha track detectors (ATDs), CR-39 solid-state nuclear track detectors, and Electret ion chambers (EICs).	3–12 Months	Most commonly used method for calculating the three-month annual mean concentration [5,53,61].
Short-Term Radon Monitors	Activated charcoal canisters and electronic radon detectors	2–7 Days	Preliminary screening or locations where long-term testing is not feasible [41,44].
Continuous Monitoring	Continuous radon monitors (CRMs), real-time electronic sensors.	48 h to several weeks	Assessing hourly and diurnal fluctuations of radon levels, evaluating the performance of mechanical ventilation systems, and examining occupancy effects [46,56].
Stratified Sampling	Multi-story and multi-room detector grids	Variable (by device)	Characterizing ground-contact versus upper-floor concentrations [52,62].
Seasonal Variations	Repeated seasonal deployments (Winter vs. Summer)	Multi-phase (Seasonal)	Assessing the “stack effect” and enhancing estimates of the annual mean [5,63].
Statistical Modeling	Modeling with the log-normal distribution and parameter estimation	N/A (Data-driven)	Using geometric means to depict exposure risk distributions [51].
National Surveys	Policy-based reporting, standardized national surveys	Longitudinal	Assessing trends in national testing practices and compliance with policy guidelines [44,65].
Systematic Reviews	Literature review, meta-analysis of existing data	Secondary data	Gathering radon concentration determinants and concentration ranges from existing research [43,49].

## Data Availability

No new data were created or analysed in this study.

## Supplementary Materials

The following supporting information can be downloaded at: https://www.mdpi.com/article/10.3390/ijerph23060712/s1, Table S1 (Supplementary S1): Radon Studies in Educational Settings. Table S2 (Supplementary S2): PRISMA 2020 Checklist.

## References

[1] Pirsaheb M., Najafi F., Khosravi T., Hemati L. (2013). A systematic review of radon investigations related to public exposure in Iran. Iran. Red. Crescent Med. J..

[2] Giraldo-Osorio A., Ruano-Ravina A., Pérez-Ríos M., Varela-Lema L., Barros-Dios J.M., Arias-Ortiz N.E. (2021). Residential Radon in Manizales, Colombia: Results of a Pilot Study. Int. J. Environ. Res. Public Health.

[3] Vengosh A., Coyte R.M., Podgorski J., Johnson T.M. (2022). *A* critical review on the occurrence and distribution of the uranium- and thorium-decay nuclides and their effect on the quality of groundwater. Sci. Total Environ..

[4] Banzon T.M., Greco K.F., Li L., Mukharesh L., Vieira C.L.Z., Steiner M.K., Hauptman M., Ratchataswan T., Koutrakis P., Phipatanakul W. (2023). Effect of radon exposure on asthma morbidity in the School Inner-City Asthma study. Pediatr. Pulmonol..

[5] Branco P.T., Nunes R.A., Alvim-Ferraz M.C., Martins F.G., Sousa S.I. (2016). Children’s Exposure to Radon in Nursery and Primary Schools. Int. J. Environ. Res. Public Health.

[6] Kwan W.S., Nikezic D., Roy V.A.L., Yu K.N. (2020). Multiple Stressor Effects of Radon and Phthalates in Children: Background Information and Future Research. Int. J. Environ. Res. Public Health.

[7] Kalisa E., Clark M.L., Ntakirutimana T., Amani M., Volckens J. (2023). Exposure to indoor and outdoor air pollution in schools in Africa: Current status, knowledge gaps, and a call to action. Heliyon.

[8] Oliveira M., Slezakova K., Delerue-Matos C., Pereira M.C., Morais S. (2019). Children environmental exposure to particulate matter and polycyclic aromatic hydrocarbons and biomonitoring in school environments: A review on indoor and outdoor exposure levels, major sources and health impacts. Environ. Int..

[9] Sadeghi S., Hajizadeh Y., Pirsaheb M., Teiri H., Sharafi K. (2025). Assessment of indoor radon exposure in kermanshah’s educational facilities, and its determinants, health risks, and mitigation strategies. Sci. Rep..

[10] Bouvard V., Baan R., Straif K., Grosse Y., Secretan B., El Ghissassi F., Benbrahim-Tallaa L., Guha N., Freeman C., Galichet L. (2009). A review of human carcinogens—Part B: Biological agents. Lancet Oncol..

[11] International Agency for Research on Cancer (IARC) (2026). IARC Monographs on the Identification of Carcinogenic Hazards to Humans. https://monographs.iarc.who.int/list-of-classifications.

[12] Loomis D., Guha N., Hall A.L., Straif K. (2018). Identifying occupational carcinogens: An update from the IARC Monographs. Occup. Environ. Med..

[13] Frumkin H., Samet J.M. (2001). Radon. https://acsjournals.onlinelibrary.wiley.com/doi/abs/10.3322/canjclin.51.6.337.

[14] Torres-Durán M., Barros-Dios J.M., Fernández-Villar A., Ruano-Ravina A. (2014). Residential radon and lung cancer in never smokers. A systematic review. Cancer Lett..

[15] Bennett W.D., Zeman K.L. (2004). Effect of body size on breathing pattern and fine-particle deposition in children. J. Appl. Physiol..

[16] Ginsberg G.L., Asgharian B., Kimbell J.S., Ultman J.S., Jarabek A.M. (2008). Modeling approaches for estimating the dosimetry of inhaled toxicants in children. J. Toxicol. Environ. Health Part A.

[17] Hall E.J., Giaccia A.J. (2019). Radiobiology for the Radiologist.

[18] Azzam E.I., Jay-Gerin J.P., Pain D. (2012). Ionizing radiation-induced metabolic oxidative stress and prolonged cell injury. Cancer Lett..

[19] Banzon T.M., Jung Y.S., Greco K.F., Li L., Nadeau K., Permaul P., Koutrakis P., Gaffin J.M., Phipatanakul W. (2025). Biomarkers of inflammation associated with radon exposure in the School Inner-City Asthma Study (SICAS). J. Allergy Clin. Immunol..

[20] Tong J., Qin L., Cao Y., Li J., Zhang J., Nie J., An Y. (2012). Environmental radon exposure and childhood leukemia. Journal of toxicology and environmental health. Part B, Critical reviews. J. Toxicol. Environ. Health B Crit. Rev..

[21] World Health Organization (WHO) (2021). More Countries Act Against Exposure to Radon and Associated Cancer Risks. https://www.who.int/news/item/04-02-2021-more-countries-act-against-exposure-to-radon-and-associated-cancer-risks#:~:text=Globally%2C%20in%202019%2C%20residential%20radon,people%20who%20have%20never%20smoked.

[22] Yusuf R., Rathebe P.C. (2026). Health Implications of Radon Exposure Among Children: A Systematic Review. Children.

[23] Ambrosino F., La Verde G., Oliva R., Hanfi M.Y., Sarno A., e Pugliese M. (2025). Indoor radon risk assessment in education buildings from kindergarten to high school in the Campania region. Front. Built Env..

[24] Nazaroff W.W., Doyle S.M. (1985). Radon entry into houses having a crawl space. Health Phys..

[25] Richter E.D., Neeman E., Fischer I., Berdugo M., Westin J.B., Kleinstern J., Margaliot M. (1997). Radon exposures in a Jerusalem public school. Environ. Health Perspect..

[26] Dovjak M., Vene O., Vaupotič J. (2022). Analysis of Ventilation Efficiency as Simultaneous Control of Radon and Carbon Dioxide Levels in Indoor Air Applying Transient Modelling. Int. J. Environ. Res. Public Health.

[27] Zhukovsky M., Vasilyev A., Onishchenko A., Yarmoshenko I. (2018). Review of Indoor Radon Concentrations in Schools and Kindergartens. Radiat. Prot. Dosim..

[28] Appleton J.D. (2007). Radon: Sources, Health Risks, and Hazard Mapping. AMBIO J. Hum. Environ..

[29] Kelly-Reif K., Bertke S.J., Rage E., Demers P.A., Fenske N., Deffner V., Kreuzer M., Samet J., Schubauer-Berigan M.K., Tomasek L. (2023). Radon and lung cancer in the pooled uranium miners analysis (PUMA): Highly exposed early miners and all miners. Occup. Environ. Med..

[30] Nunes L.J.R., Curado A., Lopes S.I. (2023). The Relationship between Radon and Geology: Sources, Transport and Indoor Accumulation. Appl. Sci..

[31] Otton J.K. (1992). The Geology of Radon—U.S. Department of the Interior. https://pubs.usgs.gov/gip/7000018/report.pdf.

[32] Schumann R.R., Owen D.E., Asher-Bolinder S., Gates A.E., Gundersen L.C.S. (1992). Effects of weather and soil characteristics on temporal variations in soil-gas radon concentrations. Geologic Controls on Radon.

[33] Eriksen M.B., Frandsen T.F. (2018). The impact of patient, intervention, comparison, outcome (PICO) as a search strategy tool on literature search quality: A systematic review. J. Med. Libr. Assoc. JMLA.

[34] Frandsen T.F., Bruun Nielsen M.F., Lindhardt C.L., Eriksen M.B. (2020). Using the full PICO model as a search tool for systematic reviews resulted in lower recall for some PICO elements. J. Clin. Epidemiol..

[35] Methley A.M., Campbell S., Chew-Graham C., McNally R., Cheraghi-Sohi S. (2014). PICO, PICOS and SPIDER: A comparison study of specificity and sensitivity in three search tools for qualitative systematic reviews. BMC Health Serv. Res..

[36] RAYYAN (2025). Getting Started with Rayyan—A Quick Start Guide.

[37] Landrigan P.J., Kimmel C.A., Correa A., Eskenazi B. (2004). Children’s health and the environment: Public health issues and challenges for risk assessment. Environ. Health Perspect..

[38] Sackett D.L., Rosenberg W.M., Gray J.A., Haynes R.B., Richardson W.S. (1996). Evidence based medicine: What it is and what it isn’t. BMJ.

[39] Grimes D.A., Schulz K.F. (2002). Bias and causal associations in observational research. Lancet.

[40] Mann C.J. (2003). Observational research methods. Research design II: Cohort, cross sectional, and case-control studies. Emerg. Med. J. EMJ.

[41] Kitto M. (2014). Radon testing in schools in New York State: A 20-year summary. J. Environ. Radioact..

[42] Onishchenko A., Malinovsky G., Vasilyev A., Zhukovsky M. (2017). Radon Measurements in Kindergartens in Ural Region (Russia). Radiat. Prot. Dosim..

[43] Salonen H., Salthammer T., Alapieti T., Shirazi A., Mikkola R., Morawska L. (2025). Indoor radon concentrations in European kindergartens and other educational facilities. Environ. Int..

[44] United States Environmental Protection Agency (US EPA) (2025). Radon Measurement in Schools. Revised Edition. https://www.epa.gov/system/files/documents/2025-06/2025-radon_measurement_in_schools.pdf.

[45] Maheso A.M., Bezuidenhout J., Newman R.T. (2023). Indoor Radon Levels in Homes and Schools in the Western Cape, South Africa-Results from a Schools Science Outreach Initiative and Corresponding Model Predictions. Int. J. Environ. Res. Public Health.

[46] Rey J.F., Cesari M., Goyette Pernot J. (2025). Considering the presence of users while assessing indoor radon levels in public schools on short-term periods of investigation. J. Phys. Conf. Ser..

[47] Kullab M.K., Al-Bataina B.A., Ismail A.M., Abumurad K.M., Ghaith A. (1997). Study of radon-222 Concentration levels inside kindergartens in Amman. Radiat. Meas..

[48] Maged A.F. (2006). Radon concentrations in elementary schools in Kuwait. Health Phys..

[49] Astuti A., Tejamaya M. An Overview of Radon Concentrations and Risk Factors in Elementary School Classrooms: Systematic Literature Review 2010–2020. Proceedings of the 11th Annual International Conference on Industrial Engineering and Operations Management.

[50] Branco P.T.B.S., Martin-Gisbert L., Sá J.P., Ruano-Raviña A., Barros-Dios J., Varela-Lema L., Sousa S.I.V. (2023). Quantifying indoor radon levels and determinants in schools: A case study in the radon-prone area Galicia-Norte de Portugal Euroregion. Sci. Total Environ..

[51] Kouroukla E., Gooding T.D. (2024). Distribution of radon in large workplaces: An analysis performed on radon levels measured in UK schools. J. Radiol. Prot. Off. J. Soc. Radiol. Prot..

[52] Yao M., Ding K., Tang X., Wu Y., Song Y., Liu S., Bai B., Zhang L., Ma Y. (2024). Analysis and Monitoring of Indoor Radon Concentrations of 37 Kindergartens—Beijing Municipality, China, 2023. China CDC Wkly..

[53] Kojo K., Kurttio P. (2020). Indoor Radon Measurements in Finnish Daycare Centers and Schools—Enforcement of the Radiation Act. Int. J. Environ. Res. Public Health.

[54] Synnott H., Hanley O., Fenton D., Colgan P.A. (2006). Radon in Irish schools: The results of a national survey. J. Radiol. Prot. Off. J. Soc. Radiol. Prot..

[55] Loffredo F., Opoku-Ntim I., Meo G., Quarto M. (2022). Indoor Radon Monitoring in Kindergarten and Primary Schools in South Italy. Atmosphere.

[56] Davis E.A., Ou J.Y., Chausow C., Verdeja M.A., Divver E., Johnston J.D., Beard J.D. (2020). Associations Between School Characteristics and Classroom Radon Concentrations in Utah’s Public Schools: A Project Completed by University Environmental Health Students. Int. J. Environ. Res. Public Health.

[57] Titipornpun K., Gimsa J., Bhongsuwan T., Kongchouy N., Titipornpun A. (2015). Radon Concentration Measurements in Secondary Schools, Surat Thani Province, Southern Thailand. Conf. Int. J. Arts Sci..

[58] Vaupotič J., Bezek M., Kávási N., Ishikawa T., Yonehara H., Tokonami S. (2012). Radon and thoron doses in kindergartens and elementary schools. Radiat. Prot. Dosim..

[59] Poffijn A., Uttenhove J., Drouget B., Tondeur F. (1994). The Radon Problem in Schools and Public Buildings in Belgium. Radiat. Prot. Dosim..

[60] López-Pérez M., Hernández F., Díaz J.P., Salazar-Carballo P.A. (2022). Determination of the indoor radon concentration in schools of Tenerife (Canary Islands): A comparative study. Air Qual. Atmos. Health.

[61] Dias D.C.S., da Silva N.C., Silva W.L., Rodrigues M.V. (2021). Indoor radon assessment in kindergartens: Towards a national action plan. Braz. J. Radiat. Sci..

[62] Al Zabadi H.A., Mallah K., Saffarini G. (2015). Indoor exposure assessment of radon in the elementary schools, Palestine. Iran. J. Radiat. Res..

[63] Bem H., Bem E.M., Krawczyk J., Płotek M., Janiak S., Mazurek D. (2013). Radon concentrations in kindergartens and schools in two cities: Kalisz and Ostrów Wielkopolski in Poland. J. Radioanal. Nucl. Chem..

[64] Birovljev A., Strand T., Heiberg A. (1998). Radon Concentrations in Norwegian Kindergartens: Survey Planning and Preliminary Results. https://www.osti.gov/etdeweb/servlets/purl/359845.

[65] Everett J.S., Foster S., Berens A.S. (2014). Radon Testing Status in Schools by Radon Zone and School Location and Demographic Characteristics: United States, 2014. J. Sch. Nurs. Off. Publ. Natl. Assoc. Sch. Nurses.

[66] Shergill S., Forsman-Phillips L., Nicol A.M. (2021). Radon in Schools: A Review of Radon Testing Efforts in Canadian Schools. Int. J. Environ. Res. Public Health.

[67] Azara A., Dettori M., Castiglia P., Piana A., Durando P., Parodi V., Salis G., Saderi L., Sotgiu G. (2018). Indoor Radon Exposure in Italian Schools. Int. J. Environ. Res. Public Health.

[68] Darby S., Hill D., Auvinen A., Barros-Dios J.M., Baysson H., Bochicchio F., Deo H., Falk R., Forastiere F., Hakama M. (2005). Radon in homes and risk of lung cancer: Collaborative analysis of individual data from 13 European case-control studies. BMJ.

[69] Coretchi L., Ene A., Virlan S., Gincu M., Ababii A., Capatina A., Overcenco A., Sargu V. (2023). Children’s Exposure to Radon in Schools and Kindergartens in the Republic of Moldova. Atmosphere.

[70] Poulin P., Leclerc J.M., Dessau J.C., Deck W., Gagnon F. (2012). Radon measurement in schools located in three priority investigation areas in the province of Quebec, Canada. Radiat. Prot. Dosim..

