# Supplementary material for: Indoor Radon Exposure Among Schoolchildren: A Systematic Review of Risk Factors
Source: Int J Environ Res Public Health. 2026 May 27;23(6):712. doi: 10.3390/ijerph23060712 (PMC13299810; doi:10.3390/ijerph23060712)
Supplement: Supplementary file 1 [file ijerph-23-00712-s001.zip › Supplementary Table S1 V1 - final.pdf]

Table S1. Radon Studies in Educational Settings. Note: Daycare/Kindergarten (0–6 years), Primary schools (7–12 years), or Secondary schools (13–18 years).

Authors	Country	Age Group	Study Type	NOS	Avg. Radon (Bq/m3)	Measurement Method	Outcomes
Branco et al. [5]	Portugal	Daycare & Kindergarten	Env. Monitor	High	88–111	Continuous Monitoring (AlphaGuard)	Average radon exposure hovers around the WHO reference level (100 Bq/m³), which could be attributed to the ventilation system in the classrooms.
Sadeghi et al. [9]	Iran	Daycare & Primary	Cross-Sectional	Mod-High	37.1 (K); 30.9 (S)	Passive CR-39 Detectors (90 days)	Values though below the WHO 100 Bq/m³ and US EPA 148 Bq/m³ thresholds, building materials were a factor.
Richter et al. [25]	Israel	Primary & Secondary	Case Study	Moderate	300–10,000	Active Pylon Monitoring & Charcoal	Soil depressurization reduced levels to <75 as a mitigation measure.
Kitto [41]	USA	Primary	Longitudinal	Moderate	< 148	Passive Alpha Track (Long-term)	The average radon in the classrooms is lower than the regional reference of 148 Bq/m³ due to HVAC operations.
Onishchenko et al. [42]	Russia	Primary & Secondary	Cross-Sectional	Moderate	49	Passive Adsorption (Charcoal) & Track Etch	High variability; peak reached 800 Bq/m³.
Salonen et al. [43]	Europe	Mixed: Daycare-Secondary	Lit. Review	Mod-High	6–1475	Multiple (Meta-analysis of SSNTD/Active)	A significant number exceeded the WHO 100 Bq/m³ due to inadequate sealing and ventilation.
US EPA [44]	USA	All school ages	Nat. Survey	High	148	Activated Charcoal / ATD (EPA Protocol)	1 in 5 schools are above 148 due to high-risk Zone 1.
Maheso et al. [45]	South Africa	Primary	Cross-Sectional	Moderate	38	CR-39 Passive Detectors	Only 4% exceeded WHO 100 Bq/m³, possibly most classrooms on concrete slabs.
Rey et al. [46]	Switzerland	Primary & Secondary	Methodological	Mod-High	(Some exceedance of the EU 300 Bq/m³)	Continuous (Active) vs. CR-39 (Passive)	Short-term testing impractical for regulation with probability of exceedance of 300 Bq/m³.
Kullab et al. [47]	Jordan	Primary & Secondary	Env. Survey	Mod-High	76.8	CR-39 Etched Track Detectors	Average level lower than the WHO 100 Bq/m³, influenced by building age/ventilation habits (open vs close windows).
Maged [48]	Kuwait	Primary & Secondary	Cross-Sectional	Moderate	16–19	CR-39 Solid State Track	Exposure below WHO/EPA/ICRP

Astuti & Tejamaya [49]	Global	All school ages	Lit. Review	Mod-High	119	Review of ATD/Charcoal/Active Methods	Annual dose exceeded UNSCEAR 1.15mSv/y.
Branco et al. [50]	Spain	Mixed: primary & Secondary	Cross-Sectional	High	332	CR-39 Etched Track Detectors (Long-term)	32.7% exceeded the EU limit (300 Bq/m3) due partly to granite soil.
Kouroukla & Gooding [51]	UK	Primary & Secondary	Stat. Modeling	Mod-High	300	Modeling based on CR-39 field data	Distribution parameters more accurate than mean, aligned with the EU reference level (300 Bq/m³).
Yao et al. [52]	China	Primary	Env. Survey	Mod-High	84.3	SSNTD (CR-39) Passive Detectors	2.4% exceeded 200 Bq/m^3, often at ground floor.
Kojo & Kurttio [53]	Finland	Daycare & Secondary	Cross-Sectional	High	85 (DC); 82 (S)	Alpha Track (Finish Radiation Authority)	Extremely high peaks (up to 4205) due to soil porosity.
Synnott et al. [54]	Ireland	Primary	Cross-Sectional	High	93	CR-39 Etched Track (National Protocol)	Average effective dose of 0.3 mSv/year linked to wall and floor radium contents.
Loffredo et al. [55]	Italy	Primary & Secondary	Cross-Sectional	High	98	CR-39 Passive Solid-State Nuclear Track	30% exceeded the WHO 100 Bq/m^3, often in old school buildings; newer schools had lower levels.
Davis et al. [56]	USA	Mixed: K 1-12	Cross-Sectional	High	31.39 (GM)	Alpha Track Detectors (ATD)	2% of classrooms met or exceeded US EPA 148 Bq/m^3.
Titipornpun et al. [57]	Thailand	Primary & Secondary	Cross-Sectional	Moderate	19 (GM)	CR-39 Etched Track Detectors	Levels generally low (2–167) owing to cross-ventilation.
Vaupotic et al. [58]	Slovenia	Daycare & primary	National Survey	Moderate	145–794	Scintillation Cells & CR-39	High levels above EU reference range of 300 Bq/m^3 are attributed to regional geology.
Poffijn et al. [59]	Belgium	Primary	Cross-Sectional	Mod-High	> 400	CR-39 Detectors (Winter/Summer avg)	Elevated levels in radon-prone regions, well above WHO 100 Bq/m^3.
López-Pérez et al. [60]	Spain	Primary	Quant. Comp.	Mod-High	73.5	Electret Ion Chambers (EIC)	Estimated annual dose within the limit of 1mSv/year.
Dias et al. [61]	Brazil	Primary	Cross-Sectional	High	83	CR-39 Passive Detectors	4% exceeded 300 Bq/m^3 limit due to cracks and openings in the floor.
Al Zabadi et al. [62]	Palestine	Primary & Secondary	Cross-Sectional	Moderate	40.42	CR-39 Track Etch Detectors	Radon within ICRP 300 Bq/m^3 reference, though higher at the floor level.
Bem et al. [63]	Poland	Primary	Env. Survey	Mod-High	46–48.9	CR-39 Etched Track Detectors	Results < ICRP 300 Bq/m^3 reference levels.
Birovljev et al. [64]	Norway	Primary	Cross-Sectional	Moderate	69	Track Etch Detectors (Seasonal correction)	6% of classrooms exceeded 200 due stack effect.

Everett et al. [65]	USA	All school ages	Nat. Survey	Moderate	46–148	Activated Charcoal / Alpha Track (Survey)	Low awareness, with only 46% of schools reported to be tested.
Shergill et al. [66]	Canada	All school ages	Qual. Enquiry	Moderate	N/A	Alpha Track (Review of testing data)	Fragmented testing across provinces, with results exceeding the regulatory reference of 200 Bq/m^3 in Canada.
Azara et al. [67]	Italy	Primary & Secondary	Env. Monitor	High	91.6	CR-39 Etched Track Detectors	The average radon is lower than the WHO reference level of 100 Bq/m^3, despite wide variation of radon levels in the foundations.
Coretchi et al. [69]	Moldova	Daycare & Kindergarten	Quant. Survey	Mod-High	148.4; 127.6	SSNTD (CR-39) Passive Monitoring	Kindergarten levels are lower than other educational settings, yet both are lower than EU 300 Bq/m^3
Poulin et al. [70]	Canada	Primary	Env. Survey	Mod-High	56	Alpha Track Detectors (3-month avg)	Most classrooms below 200 Bq/m^3 even in high-risk areas.
