# Supplementary material for: Indoor Radon Exposure Among Schoolchildren: A Systematic Review of Risk Factors
Source: Int J Environ Res Public Health. 2026 May 27;23(6):712. doi: 10.3390/ijerph23060712 (PMC13299810; doi:10.3390/ijerph23060712)
Supplement: Supplementary file 1 [file ijerph-23-00712-s001.zip › ijerph-4229294-supplementary 2 - Prisma Checklist - final.pdf]

Supplementary 2. TABLE S2: PRISMA 2020 Checklist

Section and Topic	Item #	Checklist item	Location where item is reported
**TITLE**		**Indoor Radon Exposure among Schoolchildren: A Systematic Review of Risk Factors**	
Title	1	The report is identified as a systematic review in the title.	Lines 2-3.
**ABSTRACT**			
Abstract	2	The Abstract includes the study aim/objective, methods (databases, GRADE), results (n=32 studies), and public health implications.	Within the Abstract
**INTRODUCTION**			
Rationale	3	The study highlights radon as a Group 1 carcinogen with exposure burden among schoolchildren. In addition, children face vulnerabilities due to the distinct challenges posed by their physiological and behavioral traits.	Within the Introduction section
Objectives	4	Research questions: methods of radon measurement, exposure levels, and the risk factors for exposure.	At the end of the Introduction section
**METHODS**			
Eligibility criteria	5	Inclusion/exclusion criteria focused on:<br>• Population (<18 years)<br>• Study design (quantitative)<br>• Setting (daycare centers, kindergartens, schools)<br>• Outcomes (radon concentration as well as the risk factors)	Section 2.4.1.
Information sources	6	Databases: Scopus, Web of Science, PubMed/MEDLINE, Cochrane Library, Google Scholar.<br>Search cutoff: January 28, 2026.	Sections 2.3. and 2.4.2
Search strategy	7	The search strategy encompassed the following terms: ("Radon" OR "Radon gas" OR "222 Rn") AND ("Classrooms", "Schools" OR "Indoor" OR "Educational Facilities") AND ("Schoolchildren" OR "Children", "Pediatrics" OR "Radon in Children" OR "Children Exposure") AND ("Risks" OR "Risk Factors"). A comprehensive search was conducted across multiple databases up until January 28, 2026, using the term "Radon," which initially yielded 127,696 results.	Sections 2.3. and 2.4.2
Selection process	8	Three independent reviewers were involved in the study. Screening and eligibility steps were described in the PRISMA Flow Chart (Figure 1).	Section 2.4.1. and the PRISMA Flow Chart (Figure 1).
Data collection process	9	Three reviewers extracted data. No automation tools were used.	Section 2.4.1.
Data items	10a	Primary data outcomes include: indoor radon concentration (Bq/m³). Secondary data outcome: risk factors (geology,floor level, ventilation patterns, etc).	Section 2.4.1.
	10b	Other extracted variables include geographic location, sample size, and measurement duration.	Section 2.4.1.

Supplementary 2. TABLE S2: PRISMA 2020 Checklist

Section and Topic	Item #	Checklist item	Location where item is reported
Study risk of bias assessment	11	The risk of bias was assessed with the Newcastle-Ottawa Scale (NOS).	Section 2. 4.4. and Table 3.
Effect measures	12	Effects were measured with descriptive statistics (mean, SD, radon concentration levels).	Methods and Results sections.
Synthesis methods	13a	Studies were categorized by exposure features and methodological quality.	Sections 2.4.1. – 5.
	13b	Study sample size was assumed, considering a sample size of at least 300 participants as 'High-Yield Quality' for robustness. Large-scale studies over 300 participants include multi-regional research, such as in Ireland (3,500 schools), Finland (3,000 facilities). Studies with fewer than 300 participants, such as Maheso et al. (2023), are considered small samples, assuming 20 pupils per classroom.	Table 2.
	13c	Use of tables (Tables 1–3), Figure 2, and structured narrative synthesis in the Discussion section.	Tables 1 – 3, Figure 2, and the Discussion section.
	13d	Narrative synthesis was used due to heterogeneity. No meta-analysis conducted.	Results section.
	13e	The study's heterogeneity was explored qualitatively (geologic diversities, measurement duration, methodology).	Sections 2.4.3. and 2.4.4.
	13f	Not applicable due to no meta-analysis conducted.	N/A
Reporting bias assessment	14	The study's Limitations.	Section 4.4.2.
Certainty assessment	15	The GRADE framework was applied, modified for the environmental health context. Also, the Newcastle-Ottawa Scale (NOS).	Section 2.4.3.
**RESULTS**			
Study selection	16a	The full screening process was documented. Initially, 127,696 studies were narrowed to 32.	PRISMA Flow Chart (Figure 1).
	16b	Excluded studies include those conducted exclusively within the workplace or higher education centers (polytechnics, universities, etc.), and adult population studies targeting cohorts older than 18 years.	Section 2.4.1.
Study characteristics	17	The list of all the included 32 studies and their characteristics is provided in Table 1 (Supplementary 1).	Table 1 (Supplementary 1).
Risk of bias in studies	18	A detailed evaluation using the Newcastle-Ottawa Scale was presented.	Section 2.4.4. and Table 3.
Results of individual studies	19	Radon levels, statistical characteristics, and outcome were reported for each of the 32 included studies.	Table 1 (Supplementary 1).
Results of	20a	A narrative synthesis of radon exposure patterns and regional variability in relation to risk factors was presented in the Results section.	Results section.

Supplementary 2. TABLE S2: PRISMA 2020 Checklist

Section and Topic	Item #	Checklist item	Location where item is reported
syntheses	20b	No quantitative pooling or meta-analysis conducted.	N/A
	20c	No quantitative pooling or meta-analysis conducted.	N/A
	20d	No quantitative pooling or meta-analysis conducted.	N/A
Reporting biases	21	The risk of bias was discussed in Section 2.4.4.	Section 2.4.4.
Certainty of evidence	22	A GRADE-based certainty evaluation is provided in Section 2.4.3.	Section 2.4.3.
**DISCUSSION**			
Discussion	23a	The general interpretation of the study's results was contextualized globally, guided by the WHO (100 Bq/m³), EU (300 Bq/m³), and US EPA (148 Bq/m³) radon thresholds.	Discussion section.
	23b	Limitations of the evidence in the review include variability in measurement durations, study site selection (soil geology), measurement tool bias, and study design constraints.	Section 4.4.2.
	23c	During the review of included studies, it was noted that Vaupotic et al. (2012) [58] included radon and thorium measurements across educational settings. Hence, this study did not focus exclusively on radon measurement.	Table 3.
	23d	Policy and public health recommendations are provided.	Highlights and in lines 479-488
**OTHER INFORMATION**			
Registration and protocol	24a	University of Johannesburg Faculty of Health Sciences Research Ethics Committee (REC; Clearance No. REC-2969-2024), and the review was registered with the International Prospective Register of Systematic Reviews (PROSPERO), under registration number CRD420261291315.	Section 2.2.
	24b	The protocol of the review can be accessed through the International Prospective Register of Systematic Reviews (PROSPERO), under registration number CRD420261291315.	Section 2.2.
	24c	No amendments to the information provided in the protocol.	N/A
Support	25	Describe sources of financial or non-financial support for the review, and the role of the funders or sponsors in the review.	Funders mentioned in line 636 and 637 played no role in the support of the literature review.
Competing interests	26	No competing interests of review authors.	N/A
Availability of data, code and other materials	27	The protocol of the review can be accessed through the International Prospective Register of Systematic Reviews (PROSPERO), under registration number CRD420261291315. Data extracted from included studies are provided in Supplementary 1. Schematic summary of Indoor Radon Exposure in Educational Settings is included as Figure 2.	Section 2.2, Table 1 (Supplementary 1), and Figure 2.

Supplementary 2. TABLE S2: PRISMA 2020 Checklist

From: Page MJ, McKenzie JE, Bossuyt PM, Boutron I, Hoffmann TC, Mulrow CD, et al. The PRISMA 2020 statement: an updated guideline for reporting systematic reviews. BMJ 2021;372:n71. doi: 10.1136/bmj.n71. This work is licensed under CC BY 4.0. To view a copy of this license, visit https://creativecommons.org/licenses/by/4.0/
